# Mineralocorticoid receptor antagonists for the treatment of heart failure and kidney disease: a state-of-the-art review

**DOI:** 10.1007/s10741-026-10636-0

**Published:** 2026-05-26

**Authors:** Harriette G. C. Van Spall, Orly Vardeny

**Affiliations:** 1 Faculty of Health Sciences, McMaster University, 20 Copeland Avenue, Suite C3-117, Hamilton, ON L8S 4L8 Canada; 2 Population Health Research Institute, Hamilton, Canada; 3 Department of Medicine, University of Minnesota, Minneapolis, MN USA

**Keywords:** Benefits and risks, Heart failure, Hyperkalaemia, Mineralocorticoid receptor antagonist

## Abstract

Chronic heart failure causes significant morbidity and mortality worldwide. Mineralocorticoid receptor antagonists are pivotal in the management of heart failure with reduced ejection fraction, as demonstrated in large outcomes trials that tested the efficacy of the steroidal mineralocorticoid receptor antagonists, spironolactone and eplerenone. There is still debate regarding their use in the management of heart failure with mildly reduced ejection fraction or heart failure with preserved ejection fraction. The nonsteroidal mineralocorticoid receptor antagonist finerenone was recently shown to improve outcomes in heart failure with mildly reduced ejection fraction and heart failure with preserved ejection fraction. This review of clinical trials evaluates the efficacy and safety of steroidal and nonsteroidal mineralocorticoid receptor antagonists for the treatment of heart failure across ejection fraction categories. We discuss the benefits of mineralocorticoid receptor antagonists in treating heart failure, including in the setting of chronic kidney disease, and review the benefits of nonsteroidal mineralocorticoid receptor antagonists in patients with chronic kidney disease who are at risk of developing heart failure. We outline the effect of mineralocorticoid receptor antagonists on serum potassium levels and propose strategies for minimizing the risk of hyperkalaemia. With a focus on clinical trial evidence, this review highlights that mineralocorticoid receptor antagonists have a favourable benefit–risk in the prevention and treatment of heart failure.

## Introduction

Chronic heart failure (HF) is characterized by structural or functional impairment of ventricular ejection or filling that causes elevations in intracardiac pressures and clinical symptoms of congestion [1, 2]. Classified according to left ventricular ejection fraction (EF) – HF with reduced EF (HFrEF; HF with EF ≤ 40%), HF with mildly reduced EF (HFmrEF; HF with EF 41–49%), or HF with preserved EF (HFpEF; HF with EF ≥ 50%) [1, 3] – HF affects approximately 56.2 million individuals worldwide [4]. The prevalence of HF has risen globally due to an aging population and improved survival of both HF and its major underlying causes [4]. The risk factor profile and phenotype of HF has also changed, with increasing prevalence of HFpEF [5]. HF prevalence in the US is projected to increase by 46% between 2012 and 2030, leading to over 8 million adults with HF [6], representing 3.0% of the population, a rise from 2.4% in 2012 [6].

**Fig. 1:**
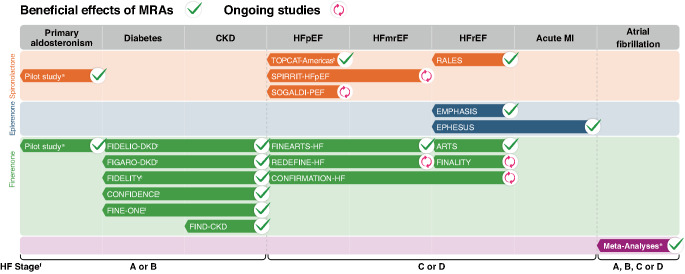
Trials that demonstrate beneficial effects of MRAs and ongoing trials across cardiovascular, kidney, and metabolic conditions. *CKD* chronic kidney disease, *HF* heart failure, *HFmrEF* heart failure with mildly reduced ejection fraction, *HFpEF* heart failure with preserved ejection fraction, *HFrEF* heart failure with reduced ejection fraction, *MI* myocardial infarction, *MRA* mineralocorticoid receptor antagonist, *nsMRA* nonsteroidal mineralocorticoid receptor antagonist, *sMRA* steroidal mineralocorticoid receptor antagonist. ^a^Reduced blood pressure. ^b^Primary endpoint not significantly reduced in TOPCAT overall. ^c^In participants with CKD, albuminuria, and type 2 diabetes. ^d^In participants with CKD, albuminuria, and type 1 diabetes;^e^Two meta-analyses: one included studies of spironolactone, eplerenone, and canrenone, the second included studies of spironolactone, eplerenone, canrenone, and finerenone; ^f^HF stages: A (at risk) = asymptomatic without structural or functional heart disease or abnormal biomarkers and with HF risk factors; B (pre-HF) = asymptomatic with structural or functional impairment of ventricular filling or ejection; C = symptomatic HF; D = advanced HF [1]

Mineralocorticoid receptor antagonists (MRAs) are considered one of four essential pillars in the management of HFrEF [7–9], with more recent evidence supporting their use in HFmrEF and HFpEF (Fig. 1) [1–3, 10–12]. Similar to sodium glucose cotransporter 2 inhibitors (SGLT2is), MRAs improve outcomes in clinically evident (Stage C) and more advanced (Stage D) HF across the EF continuum [7–9, 11, 13]. MRAs also improve outcomes in people with diabetes with chronic kidney disease (CKD), a common comorbidity that increases HF mortality but is paradoxically associated with underutilization of MRAs [14–18]. The other two pillars in HFrEF – beta-blockers and renin–angiotensin system inhibitors or angiotensin receptor/neprilysin inhibitors [1, 2, 19] – have limited evidence to support their use as first-line therapy in HFpEF, likely due to the heterogeneous and varied mechanisms of HFpEF relative to HFrEF [10].

In this narrative review, we synthesize the efficacy and safety of two distinct types of MRAs – steroidal MRAs (sMRAs) and nonsteroidal MRAs (nsMRAs) – in people with HF. We discuss the benefits of these MRAs, which differ in structure, selectivity, and potency, across EF groups and stages in HF. We also discuss their use in treating the underlying causes and comorbidities of HF. In addition, we review the side effects of these MRAs and outline an approach to maximize their tolerability.

## Effect of MRAs on clinical outcomes in patients with HF

Treatments for HF are broad, typically target cardiovascular, kidney, and metabolic mechanisms (renin–angiotensin system inhibition, SGLT2is, MRAs, beta-blockers and glucagon-like peptide-1 receptor agonists), may differ in efficacy across left ventricular EF categories [1–3, 20], and have been reviewed previously [13]. The clinical efficacy of MRAs has been demonstrated across the continuum of left ventricular EF and is summarized in Table 1 [7–9, 11, 12, 14, 15, 17, 21–28]. Guidelines strongly recommend the use of sMRAs in HFrEF, with starting doses for spironolactone of 12.5 − 25 mg once daily and a target dose of 25 − 50 mg once daily (25 mg in people with an estimated glomerular filtration rate [eGFR] of 31 − 49 mL/min/1.73^2^); and starting doses of eplerenone of 25 mg once daily, with a target dose of 50 mg once daily [1, 2, 20]. Guidelines also recommend the nsMRA, finerenone, either as an alternative to or preferred over sMRAs, in people with HFmrEF or HFpEF [20, 29, 30]. In clinical studies of finerenone in HF, initial doses were 10 − 20 mg once daily, with target doses of 20 − 40 mg once daily, depending on eGFR [11, 14, 15, 17, 27].

**Table 1:** Key MRA outcomes trials

			*N*		Key findings			
					Efficacy	Safety		
Study	Study population	Active treatment and comparator	Active	Comparator	Clinical outcomes	Hyperkalaemia	Hypokalaemia	
**HFrEF**								
RALES [7]	HFrEF (NYHA class III/IV; EF ≤ 35%) Serum creatinine ≤ 2.5 mg/dL	Spironolactone vs. placebo	822	841	Primary outcome (death from all causes): RR: 0.70 (95% CI: 0.60–0.82); *p* < 0.001 Hospitalization for HF: RR: 0.65 (95% CI: 0.54–0.77); *p* < 0.001	2% (placebo 1%; *p* = NS)^a^	NR	
EMPHASIS-HF [9]	HFrEF (NYHA class II; EF ≤ 30% or > 30–35% with QRS duration of > 130 ms) eGFR ≥ 30 mL/min/1.73 m^2^	Eplerenone vs. placebo	1,364	1,373	Primary outcome (CV death or hospitalization for HF): aHR: 0.63 (95% CI: 0.54–0.74); *p* < 0.001 CV death: aHR: 0.76 (95% CI: 0.61–0.94); *p* = 0.01 Hospitalization for HF: aHR: 0.58 (95% CI: 0.47–0.70); *p* < 0.001	11.8% (placebo 7.2%; *p* < 0.001)^b^	7.5% (placebo 11.0%; *p* = 0.002)^c^	
EPHESUS [8]	Acute MI, HFrEF^d^	Eplerenone vs. placebo	3,319	3,313	Primary outcome (CV death or hospitalization for CV events): RR: 0.87 (95% CI: 0.79–0.95); *p =* 0.002 Death from all causes: RR: 0.85 (95% CI: 0.75–0.96); *p =* 0.008	5.5% (placebo 3.9%; *p* = 0.002)^a^	8.4% (placebo 13.1; *p <* 0.001)^c^	
**HFmrEF/HFpEF**								
TOPCAT [12, 21, 22]	HFpEF (EF ≥ 45%) eGFR ≥ 30 mL/min/1.73m^2 ^ serum creatinine <2.5 mg/dL	Spironolactone vs. placebo	1,722	1,723	Primary outcome (death from CV causes, aborted cardiac arrest, or hospitalization for HF): HR: 0.89 (95% CI: 0.77–1.04); *p* = 0.14 Russia/Georgia: HR: 1.10 (95% CI: 0.79–1.51) *p* = 0.58; Americas: HR: 0.82 (95% CI: 0.69–0.98); *p* = 0.026 (*p*_interaction =_ 0.12) Hospitalization for HF: HR: 0.83 (95% CI: 0.69–0.99); *p* = 0.04	18.7% (placebo 9.1%; *p* < 0.001)^e^ Russia/Georgia: 11.6% (placebo 8.9%; *p =* 0.08); Americas: 23.8% (placebo 8.2%; *p* < 0.001)^e^	16.2% (placebo 22.9%; *p* < 0.001)^c^ Russia/Georgia: 14.7% (placebo 16.9%; *p* = 0.21); Americas:11.3% (placebo 23.6%; *p* < 0.001)^c^	
FINEARTS-HF [11, 23]	HFmrEF or/HFpEF (EF ≥ 40%; elevated level of natriuretic peptides) eGFR ≥ 25 mL/min/1.73 m^2^	Finerenone vs. placebo	3,003	2,998	Primary outcome (composite of worsening HF events [unplanned hospitalization or urgent visit for HF] or CV death): rate ratio: 0.84 (95% CI: 0.74–0.95); *p* = 0.007 Worsening HF events: rate ratio: 0.82 (95% CI: 0.71–0.94); *p =* 0.006 CV death: HR: 0.93 (95% CI: 0.78–1.11)^f^	14.6% (placebo 7.1%)^b^ HR: 2.16 (95% CI: 1.83–2.56); *p* < 0.001	5.0% (placebo 10.3%)^c^ HR: 0.46 (95% CI: 0.38–0.56); *p* < 0.001	
**Post-MI (at risk of HF)**								
CLEAR [24]	Acute MI	Spironolactone + colchicine vs. spironolactone + placebo vs. colchicine + placebo vs. placebo alone	3,537	3,525	Co-primary outcome (CV death, or new or worsening HF): HR: 0.91 (95% CI: 0.69–1.21); *p* = 0.51 Co-primary outcome (CV death, MI, stroke, or new or worsening HF: HR: 0.96 (95% CI: 0.81–1.13); *P* = 0.60	1.1% (placebo 0.6%; *p* = 0.01)^b^	NR	
**Chronic kidney disease (at risk of or with HF)**								
BARACK-D [25]	CKD	Spironolactone + usual care vs. usual care alone	677	724	Primary outcome (death, hospitalization for heart disease, stroke, HF, TIA, or PAD): HR: 1.05 (95% CI: 0.81–1.37); *p* = 0.70 Change in eGFR at 3 years: adjusted treatment effect, − 1.14 mL/min/1.73 m^2^ (95% CI: −1.92 to − 0.37) *p* = 0.004	24.7% (placebo 13.4%; *p* < 0.001)^e^	NR	
FIDELIO-DKD [15]	CKD and albuminuria associated with T2D	Finerenone vs. placebo	2,833^g^	2,841^g^	Primary outcome (kidney failure, sustained decrease in eGFR ≥ 40%, kidney-related death): HR: 0.82 (95% CI: 0.73–0.93); *p* = 0.001 CV outcomes (CV death, nonfatal MI, nonfatal stroke, hospitalization for HF): HR: 0.86 (95% CI: 0.75–0.99); *p* = 0.03	21.7% (placebo 9.8%)^b, f^	1.0% (placebo 2.2%)^f, i^	
FIGARO-DKD [17]	CKD and albuminuria associated with T2D	Finerenone vs. placebo	3,686	3,666	Primary outcome (CV death, nonfatal MI, nonfatal stroke, hospitalization for HF): HR: 0.87 (95% CI: 0.76–0.98); *p* = 0.03 Kidney outcomes (kidney failure, sustained decrease in eGFR ≥ 40%, kidney-related death): HR: 0.87 (95% CI: 0.76–1.01)^f^	13.5% (placebo 6.4%)^b, f^	1.1% (placebo 2.4%)^f, i^	
FIDELITY [14, 26]	CKD and albuminuria associated with T2D	Finerenone vs. placebo	6,519	6,507	Kidney outcomes (kidney failure, sustained decrease in eGFR ≥ 57%, or death from kidney causes): HR: 0.77 (95% CI: 0.67–0.88); *p* = 0.0002 CV outcomes: (CV death, nonfatal MI, nonfatal stroke, or hospitalization for HF): HR: 0.86 (95% CI: 0.78–0.95); *p* = 0.0018	12.0% (placebo 5.9%)^f, i^	4.8% (placebo 10.2%)^c, f^ HR: 0.46 (95% CI: 0.40–0.53)^f^	
FINE-HEART [27]	CKD and/or HFmrEF/HFpEF	Finerenone vs. placebo	9,501	9,490	Kidney outcomes (sustained decrease in eGFR to ≥ 50% from baseline, sustained decline in eGFR to < 15 mL/min/1.73 m^2^, kidney failure and death due to kidney failure): HR: 0.80 (95% CI: 0.72–0.90; *P* < 0.001) CV outcomes (CV death, HF hospitalization, MI, or stroke): HR: 0.91 (95% CI: 0.85–−0.98; *p* = 0.01)	16.5% (placebo 7.7%)^b, j^	4.8 (placebo 10.1%)^c, j^	
FINE-ONE [28]	CKD and albuminuria associated with T1D	Finerenone vs. placebo	120	122	Primary outcome (relative change in UACR over 6 months): GMR: 0.75 (95% CI: 0.65–0.87); *p* < 0.001)	10.1% (placebo 3.3%)^h^	NR	

*aHR* adjusted hazard ratio, *CI* confidence interval, *CKD* chronic kidney disease, *CV* cardiovascular, *EF* ejection fraction, *eGFR* estimated glomerular filtration rate, *HF* heart failure, *HFmrEF* heart failure with mildly reduced ejection fraction, *HFpEF* heart failure with preserved ejection fraction, *HFrEF* heart failure with reduced ejection fraction, *HR* hazard ratio, *MI* myocardial infarction, *MRA* mineralocorticoid receptor antagonist, *NR* not reported, *NS* not statistically significant, *NYHA* New York Heart Association, *PAD* peripheral artery disease, *RASi* reninangiotensin system inhibitor, *RR* relative risk, *TIA* transient ischaemic attack, *T2D* type 2 diabetes, *UACR* urine albumin-to-creatinine ratio

^a^Serum potassium ≥6.0 mmol/L; ^b^Serum potassium >5.5 mmol/L; ^c^Serum potassium <3.5 mmol/L; ^d^EF ≤40%, and HF or diabetes mellitus; ^e^Serum potassium ≥5.5 mmol/L; ^f^*P* value not stated; ^g^Excludes enrolled participants who were excluded due to GCP violations (a total of 60 across treatment groups); ^h^includes investigator-reported adverse events with the Medical Dictionary for Regulatory Activities codes hyperkalemia and blood potassium increased; ^i^Reported as an adverse event; ^j^*n* = 9,482 in the finerenone group, *n* = 9,467 in the placebo group

### Effect in patients with HFrEF

The sMRAs spironolactone and eplerenone have been shown, in well-designed phase 3 randomized controlled trials (RCTs; RALES, EMPHASIS-HF, EPHESUS), to increase survival and reduce the risk of HF hospitalizations in HFrEF and in participants with myocardial infarction (MI), left ventricular systolic dysfunction, and concomitant symptomatic HF or diabetes mellitus (Table 1) [7–9, 13], thereby earning class 1 guideline recommendations for their use in patients with symptomatic HFrEF [1, 2, 7, 9, 19, 29, 31–33].

### Effect in patients with HFmrEF/HFpEF

The evidence for sMRAs in individuals with HFmrEF and HFpEF is less robust [13]. Spironolactone did not significantly reduce the primary outcome (cardiovascular [CV] death, aborted cardiac arrest, or hospitalization for HF) in participants with HFpEF in the TOPCAT trial, although it did reduce hospitalization for HF by 17% compared with placebo (Table 1) [21]. Analysis of the TOPCAT trial showed regional differences between the Americas versus Russia and Georgia in baseline characteristics; blood pressure, potassium, and creatinine responses to spironolactone; medication adherence; and prognosis [12]. Re-analysis by subpopulation showed a nominally significant reduction in the primary outcome in the subpopulation of participants enrolled in the Americas, who had characteristics consistent with clinical HF and per-protocol study medication use (Table 1) [12, 34]. Current US-based and Japanese HF treatment guidelines provide a class 2b recommendation for use of sMRAs in individuals with HFmrEF and HFpEF, and European guidelines provide a class 2b recommendation for their use among those with HFmrEF [1, 3, 29].

Compared with spironolactone and eplerenone, the nsMRA finerenone has higher selectivity for the mineralocorticoid receptor, a shorter half-life, and differs in its activation of transcriptional cofactor [35]. In the FINEARTS-HF RCT, finerenone reduced the primary outcome of a composite of total worsening HF events and CV death compared with placebo in participants with HFmrEF or HFpEF [11]. The benefit of finerenone was observed irrespective of EF, age, and sex [36–38]. Among recipients of finerenone, long-term treatment was estimated to extend event-free survival by up to 3 years [39]. Current Japanese guidelines provide a class 2a recommendation for the use of finerenone in people with HFmrEF or HFpEF [29]. While the latest Canadian guidelines strongly recommend MRAs, they do not distinguish nsMRAs (finerenone) from sMRAs in their recommendations; however, they qualify the general recommendation for MRAs with guidance that finerenone may be prioritized over sMRA in the presence of diabetic kidney disease [20].

### Influence of baseline kidney function on treatment effect in patients with HF

HF and CKD frequently coexist [40], and impaired kidney function in HF may limit the use of MRA therapy due to concerns regarding worsening kidney function [16, 41]. Secondary analyses of MRA trials have examined the efficacy and safety of these agents in the setting of concomitant CKD, with no evidence that baseline CKD modifies efficacy or that the modest reduction in eGFR that occurs following initiation of therapy is clinically relevant [42–48].

**Fig. 2:**
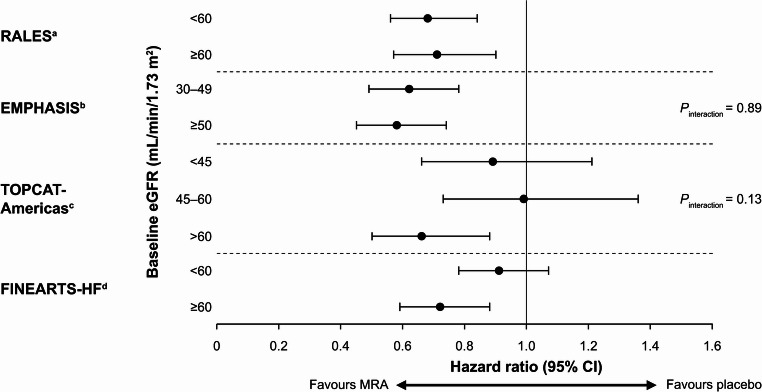
Risk of the primary trial endpoint by baseline eGFR in phase 3 trials of MRAs in patients with HF and CKD. *CI* confidence interval, *CKD* chronic kidney disease, *eGFR* estimated glomerular filtration rate, *HF* heart failure, *MRA* mineralocorticoid receptor antagonist. ^a^ Vardeny et al. 2012 [42]. Primary endpoint, all-cause deaths; ^b^Ferreira et al. 2019 [43]. Primary endpoint, composite of hospitalization for HF and CV death; ^c^Beldhuis et al. 2019 [47]. Primary endpoint, composite of hospitalization for HF, CV death, and aborted cardiac arrest; ^d^Solomon et al. 2024 [11]. Primary endpoint, worsening HF event or CV death. *P*_interaction_ values are shown for studies where available

The RALES trial (which excluded participants with serum creatinine levels > 2.5 mg/dL) demonstrated that the efficacy of spironolactone in reducing all-cause death was consistent among those with baseline eGFR of < 60 or ≥ 60 mL/min/1.73 m^2^ (Fig. 2), and was maintained even in the setting of an early 30% reduction in eGFR after spironolactone initiation [42]. Spironolactone increased median serum creatinine levels by 0.05–0.10 mg/dL compared with no change in the placebo group [7].

The EMPHASIS-HF trial excluded participants with an eGFR of < 30 mL/min/1.73 m^2^ and stratified the dose of eplerenone according to baseline eGFR (30–49 vs. ≥50 mL/min/1.73 m^2^) [9]. Eplerenone reduced the composite of hospitalization for HF and CV death in both eGFR subgroups (Fig. 2) [43], despite causing an initial decline in eGFR of 2.4 mL/min/1.73 m^2^ compared with placebo [44]. In addition, the benefit of eplerenone on survival was consistent in participants with worsening kidney function or hyperkalaemia [48]. The effect of eplerenone on the proportion of participants hospitalization for worsening kidney function was assessed as a secondary endpoint [9]. Compared with placebo, eplerenone was associated with a higher incidence of worsening kidney function and hyperkalaemia in multivariable analyses; however, the benefit of eplerenone on survival was retained in participants with worsening kidney function or hyperkalaemia [48]. Serum creatinine levels increased by 0.09 mg/dL with eplerenone and 0.04 mg/dL with placebo [9].

In a pooled analysis of data from the RALES and EMPHASIS-HF trials, 6.8% of participants had a deterioration of eGFR to < 30 mL/min/1.73 m^2^ after randomization; these patients had a lower EF and kidney function at baseline [45]. The risk of the primary outcome was higher in this group compared with participants with higher eGFR (hazard ratio [HR]: 2.49, 95% confidence interval [CI]: 2.01–3.08; *p* < 0.001). The risk reduction in the primary outcome with sMRA therapy was consistent in participants with (HR: 0.65, 95% CI: 0.43–0.99) and without (HR: 0.63, 95% CI: 0.56–0.71) a decline in eGFR to < 30 mL/min/1.73 m^2^ (*p*_interaction_ = 0.87) [45].

In the EPHESUS trial, which excluded participants with serum creatinine levels > 2.5 mg/dL at baseline [8], eGFR reduction over 24 months was greater with eplerenone compared with placebo in participants with acute MI, left ventricular dysfunction, and concomitant diabetes mellitus or HF [46]. Increases in serum creatinine concentration were modestly more pronounced with eplerenone therapy than placebo (0.06 mg/dL vs. 0.02 mg/dL at 1 year; *p <* 0.001) [8].

In a secondary analysis of TOPCAT (Americas), the efficacy and safety of spironolactone in patients with HFmrEF or HFpEF were examined across eGFR categories (≥ 60, 45–60, and 30–45 mL/min/1.73 m^2^) [47]. The benefit of spironolactone, relative to placebo, was consistent across eGFR categories (Fig. 2; *p*_interaction_ = 0.13) and when eGFR was examined as a continuous variable (*p*_interaction_ = 0.17). Study drug discontinuation for adverse effects was more common with spironolactone compared with placebo across eGFR categories, with a greater absolute incidence of spironolactone discontinuation in the lower eGFR categories (*p*_interaction_ = 0.003).

In FINEARTS-HF, the treatment effect of finerenone compared with placebo was consistent in patients with an eGFR of < 60 and ≥ 60 mL/min/1.73 m^2^ at baseline, with rate ratio 0.91 (95% CI: 0.78–1.07) and 0.72 (95% CI: 0.59–0.88), respectively [11]. A prespecified subgroup analysis of the effect of finerenone by risk category (Kidney Disease: Improving Global Outcomes: low, moderately increased, and high or very high) showed no effect of baseline kidney risk on the benefit of finerenone in patients with HFmrEF or HFpEF (*p*_interaction_ = 0.24) [49].

### Effect on CKD outcomes in patients with HF

There is evidence from RCTs to suggest that in patients with HF, MRAs reduce albuminuria [50, 51] – a marker of kidney damage – but do not delay adverse kidney events assessed as a composite of a sustained ≥ 50% eGFR decline or kidney failure (sustained eGFR of < 15 mL/min/1.73 m^2^, need for dialysis, or kidney transplantation) [51], or hospitalization for worsening kidney function [9]. The lack of an effect of MRAs on adverse kidney events in RCTs may possibly be due to the low incidence of events and competing risk of CV events in patients with HF [51]. Analysis of data from TOPCAT revealed that spironolactone reduced albuminuria by 39% overall; a lowering of albuminuria by 50% was independently associated with a reduction in HF hospitalization (HR: 0.90; *p =* 0.017) and all-cause mortality (HR: 0.91; *p* = 0.019) [50]. Similarly, finerenone reduced albuminuria, as measured by urine albumin-to-creatinine ratio (UACR), by 30% (95% CI: 25–34) over 6 months in the FINEARTS-HF trial in patients with HFmrEF/HFpEF, a benefit relative to placebo that was sustained during follow-up [51]. The risks of new-onset microalbuminuria and macroalbuminuria were reduced by 24% (HR: 0.76, 95% CI: 0.68–0.83) and 38% (HR: 0.62, 95% CI: 0.53–0.73), respectively, with finerenone compared with placebo [51]. Despite this, finerenone did not modify the composite clinical kidney outcome (sustained ≥ 50% eGFR decline, kidney failure [sustained eGFR of < 15 mL/min/1.73 m^2^], initiation of maintenance dialysis, or kidney transplantation) or the total eGFR slope [51]. The relatively low median baseline UACR levels of study participants, the low frequency of adverse kidney events in this study population, the criteria used for the kidney outcome, and the shorter duration of the trial compared with CKD trials may have limited the ability of the study to detect an effect of finerenone on kidney outcomes [51].

### Ongoing trials in patients with HF

The spironolactone in the treatment of HF trial – which was terminated after enroling about half of the planned participants and has yet to be published – did not demonstrate reduction of composite of cardiovascular death or HF hospitalizations with spironolactone in HFmrEF or HFpEF (SPIRIT-HF; NCT04727073). Ongoing studies (Fig. 1) are investigating the effects of spironolactone or eplerenone in registry participants with HFmrEF/HFpEF (SPIRRIT-HFpEF; NCT02901184) [52], and the effect of spironolactone in combination with dapagliflozin in participants with HFpEF (SOGALDI-PEF; NCT05676684) [53].

Further studies (Fig. 1) will examine the efficacy of finerenone in recently hospitalized participants with HFmrEF/HFpEF (REDEFINE-HF; NCT06008197) [54], in recently hospitalized participants with HF (regardless of EF) in combination with empagliflozin (CONFIRMATION-HF; NCT06024746) [55], and in participants with HFrEF who were previously intolerant or ineligible for sMRAs (FINALITY-HF; NCT06033950) [56].

### Effect of MRAs on other endpoints in HF trials

The effects of MRAs on blood pressure, atrial fibrillation (AF) burden, and N-terminal pro-B-type natriuretic peptide in participants with HF are summarized in Table 2 [8, 11, 21, 59, 60, 62, 63, 66]. These endpoints have prognostic significance in HF [1, 2, 57, 61, 64, 65].

**Table 2:** Association between MRAs and exploratory endpoints in HF trials

Endpoint	Summary of results
Blood pressure	Hypertension plays a key role in the development of HFpEF, and a SBP of > 140 mmHg is associated with an increase in HF events in this population [57, 58] Phase 3 trials with MRAs have demonstrated that they reduce blood pressure compared with placebo in HF populations [8, 11, 21, 59] **RALES and EMPHASIS pooled analysis** (HFrEF; *n =* 4,396) – Mean SBP change at 6 months was + 1.4 mmHg in the placebo group and − 1.2 mmHg in the MRA group (between treatment difference 2.6 mmHg; 95% CI: 1.5 − 3.6; *p* < 0.001) [59] **EPHESUS** (HFrEF; *n =* 6,632) – Mean SBP/DBP increased by 8/4 mmHg in the placebo group and by 5/3 mmHg in the eplerenone group at 1 year (*p* < 0.01) [8] **TOPCAT** (HFpEF; *n =* 3,445) – SBP at postbaseline visits was significantly lower in the spironolactone group than the placebo group (mean change from baseline at 8 months: spironolactone, − 2.7 mmHg; placebo, − 0.2 mmHg; *p* < 0.001) [21] **FINEARTS-HF** (HFmrEF/HFpEF; *n =* 6,001) – Finerenone lowered SBP compared with placebo (difference, 3.4 mmHg; 95% CI: −2.6 to − 4.2) [11] **Hu et al.**. (primary aldosteronism and hypertension; *n =* 60) – This open-label pilot study comparing the effects of finerenone and spironolactone demonstrated that mean change in daytime SBP from baseline to day 56 did not differ between the finerenone and spironolactone treatment groups [60]
AF burden	AF burden in individuals with HF and AF has been shown to predict mortality and HF events [61] Meta-analyses have demonstrated that MRAs reduce the risk of AF occurrence and AF progression in individuals with HF [62, 63]
NT-proBNP	NT-proBNP is often used as a disease biomarker to confirm the presence of HF (NT-proBNP > 125 pg/mL) and assess disease severity [1, 2] Changes in NT-proBNP are of prognostic significance and offer mechanistic insights [64], although they are less relevant than reductions in death or hospitalization Pooled analysis of RCTs in participants with HFmrEF or HFpEF demonstrated the prognostic value of NT-proBNP for predicting risk of adverse HF outcomes: risk of hospitalization for HF or CV death increased by 37% for each doubling of NT-proBNP (HR: 1.37, 95% CI: 1.34–1.41), irrespective of baseline eGFR (*p*_interaction_ = 0.42) [65] **ARTS-HF** (HFrEF; *n =* 1,066) – This phase 2b trial of participants with worsening HFrEF (requiring hospitalization for HF and treatment with intravenous diuretics), diabetes mellitus, and/or CKD, evaluated the effect of five finerenone doses (titrated to targets of 5–20 mg daily) compared with eplerenone on the percentage of participants with a > 30% decrease in NT-proBNP from baseline to day 90 (primary endpoint) [66]. The primary endpoint was achieved in 37.2% of the eplerenone group, and between 30.9% and 38.8% in the finerenone groups (depending on the dose) [66].

*AF* atrial fibrillation, *CI* confidence interval, *CKD* chronic kidney disease, *CV* cardiovascular, *DBP* diastolic blood pressure, *eGFR* estimated glomerular filtration rate, *HF* heart failure, *HFmrEF* heart failure with mildly reduced ejection fraction, *HFpEF* heart failure with preserved ejection fraction, *HFrEF* heart failure with reduced ejection fraction, *HR* hazard ratio, *MRA* mineralocorticoid receptor antagonist, *NT-proBNP* N-terminal pro-B-type natriuretic peptide, *RCT* randomized controlled trial, *SBP* systolic blood pressure

## Trials in patients at risk of HF

### Effect of MRAs following MI

In contrast to the beneficial effects of eplerenone in participants who were post-MI with HF in the EPHESUS trial [8], the use of spironolactone following MI did not reduce the incidence of HF in the CLEAR trial [24]. At baseline, < 1% of CLEAR trial participants had HF. There was no significant difference between spironolactone and placebo groups in the incidence of CV death or new or worsening HF or the composite of CV death, MI, stroke, or new or worsening HF (Table 1) [24]. Thus, there is no evidence to date to support the use of MRAs post MI in the absence of HF.

### Effect of MRAs on CV and HF outcomes in patients with CKD

Patients with CKD are at risk of HF and CV events. In the BARACK-D study in participants with Stage 3b CKD (defined as an eGFR of 30–49 mL/min/1.73 m^2^) without HF, spironolactone did not reduce CV outcomes, and safety concerns frequently resulted in spironolactone discontinuation [25]. eGFR decreased from baseline to 6 months in the spironolactone group before stabilizing for the remainder of the study [25]. Spironolactone was associated with an increased incidence of hyperkalaemia and hypotension compared with usual care. The authors concluded that spironolactone should not be used in individuals with CKD without a specific indication such as HF [25]. The study enrolled participants with CKD and minimal albuminuria (median UACR 1.5 mg/mmol), and only 75% of participants completed the study, which may limit the interpretation of the findings [25].

The beneficial effects of finerenone in diabetic kidney disease have been established [14, 15, 17, 28]. In FIDELIO-DKD, finerenone reduced kidney and CV outcomes compared with placebo in participants with CKD and albuminuria associated with type 2 diabetes (Table 1) [15]. Finerenone also reduced CV events (assessed as the primary endpoint) in FIGARO-DKD (Table 1) [17]. Data from FIGARO-DKD and FIDELIO-DKD were evaluated in a prespecified pooled analysis (FIDELITY) [14], and a further pooled analysis including FIGARO-DKD, FIDELIO-DKD, and FINEARTS-HF (FINE-HEART) was conducted [27]. In both analyses, kidney outcomes and CV events were reduced by finerenone (Table 1) [14, 27]. In the CONFIDENCE trial, which enrolled participants with CKD with albuminuria and type 2 diabetes, initial therapy with finerenone and empagliflozin reduced UACR more than with either finerenone or empagliflozin alone [18]. Current European guidelines provide a class 1a recommendation for, and the iCARDIO-alliance strongly recommends, the use of finerenone for reducing the risk of HF hospitalizations in adults with type 2 diabetes with concomitant CKD and albuminuria [3, 33].

Expanding beyond type 2 diabetes, the FINE-ONE trial recently demonstrated that finerenone reduced UACR compared with placebo by 25% over six months in participants with type 1 diabetes, CKD, and albuminuria [28]. Furthermore, the FIND‑CKD trial, which evaluated the effect of finerenone in participants with non‑diabetic CKD, ended in February 2026, following which topline results were released by the sponsor: finerenone met the primary efficacy endpoint of mean annual rate of change in eGFR from baseline to 32 months [67, 68]. While full results are pending, these findings suggest potential for the use of finerenone for slowing progression of CKD in people without underlying diabetes.

## Safety of MRAs in HF populations

### Hyperkalaemia and hypokalaemia

Aldosterone plays a central role in controlling potassium homeostasis [69]; therefore, MRAs are associated with an increased risk of hyperkalaemia [7–9, 11, 13, 21–23, 70, 71].

**Fig. 3:**
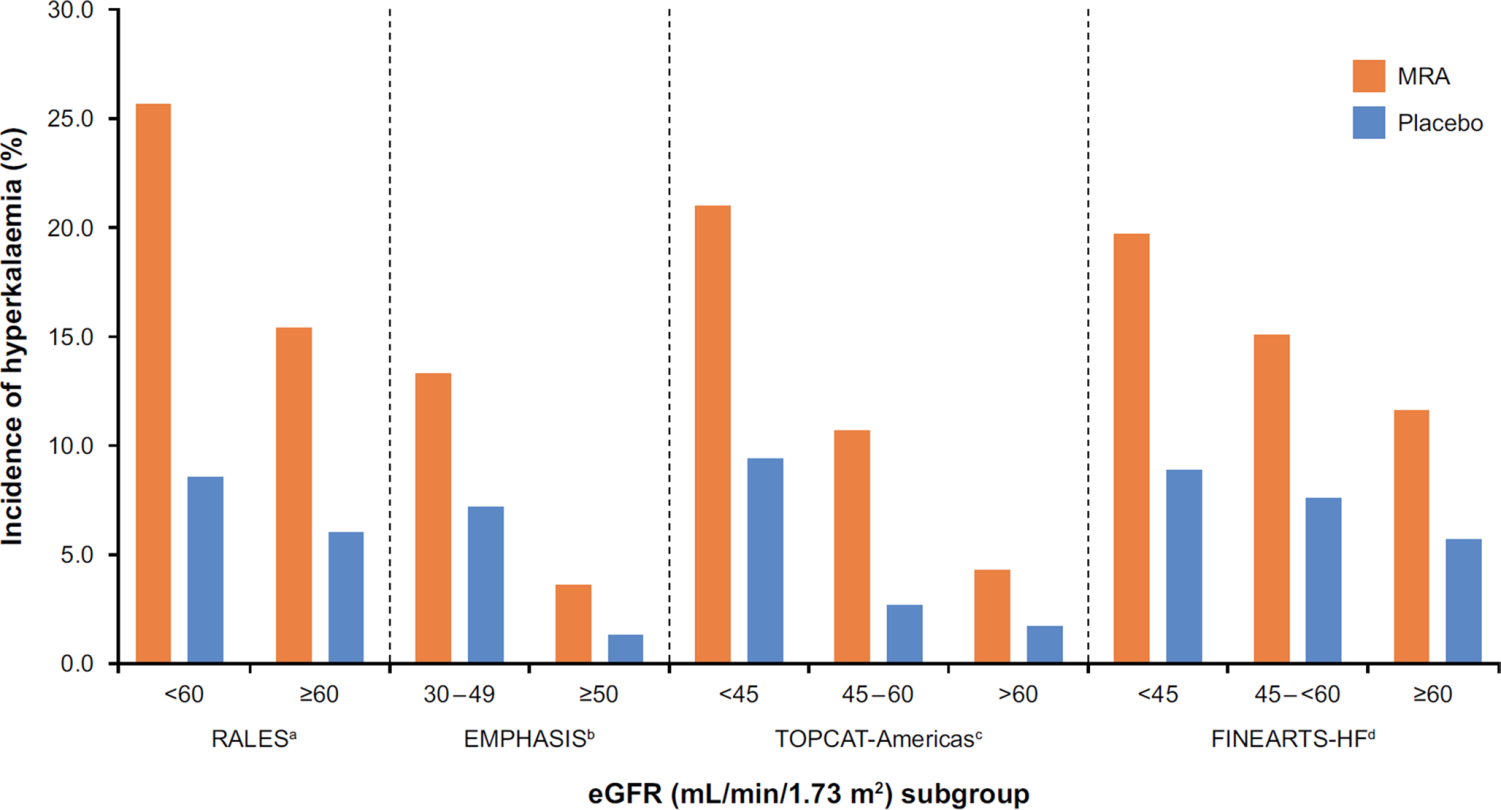
Incidence of hyperkalaemia during the trial period by baseline eGFR in phase 3 trials of MRAs in patients with HF. CKD chronic kidney disease, eGFR estimated glomerular filtration rate, HF heart failure, MRA mineralocorticoid receptor antagonist. ^a^Vardeny O et al. 2012 [42]. Hyperkalaemia defined as serum potassium >= 5.5 mmol/L or hyperkalaemia adverse event; ^b^Ferreira et al. 2019 [43]. Hyperkalaemia defined as serum potassium > 5.5 mmol/L; ^c^Beldhuis et al. 2019 [47]. Incidence shown is for the composite safety endpoint (drug discontinuation due to persistent hyperkalaemia, potassium > 5.5 mmol/L and on lowest dose, abnormal renal function [creatinine levels > 3.0 mg/dL], anaphylactic reaction, intolerance, or gynaecomastia); ^d^Mc Causland et al. 2025 [51]. Hyperkalaemia defined as serum potassium > 5.5 mmol/L

In the RALES trial, the incidence of serious hyperkalaemia (serum potassium ≥ 6.0 mmol/L) was 2% in the spironolactone group and 1% in the placebo group (Table 1; Fig. 3) [7]. Serum potassium levels were higher in the spironolactone group than in the placebo group after 1 month (4.54 vs. 4.28 mmol/L; *p* < 0.001) and remained elevated during the study [70]. The benefits associated with spironolactone were maintained despite elevation in serum at least until potassium exceeded 5.5 mmol/L [70].

In the TOPCAT trial, the incidence of hyperkalaemia (serum potassium ≥ 5.5 mmol/L) was twice as high in the spironolactone group compared with placebo, and the incidence of hypokalaemia (serum potassium < 3.5 mmol/L) was lower with spironolactone than placebo (Table 1) [21]. These estimates included patients enrolled in Russia/Georgia where a large proportion may not have had HF or received the intervention as outlined in the protocol [12, 34]. A post-hoc analysis of TOPCAT indicated that both hyperkalaemia and hypokalaemia were associated with increased risk for mortality irrespective of region [22]. In the Americas region, where the TOPCAT protocol appears to have been executed with high fidelity, spironolactone increased the risk for hyperkalaemia by more than three-fold (HR: 3.21, 95% CI: 2.46–4.20, *p <* 0.001) and reduced the risk of hypokalaemia (HR: 0.43, 95% CI: 0.34–0.55, *p <* 0.001). The treatment effect of spironolactone on the primary outcome was maintained in participants with hyperkalaemia [22].

In the EPHESUS trial, the incidence of serious hyperkalaemia (serum potassium ≥ 6.0 mmol/L) was higher with eplerenone than placebo, while the incidence of hypokalaemia (serum potassium < 3.5 mmol/L) was lower (Table 1) [8]. Compared with placebo, a higher proportion of the eplerenone group had hyperkalaemia (serum potassium > 5.5; mmol/L 11.8 vs. 7.2%; *p <* 0.001) in EMPHASIS-HF, and a lower proportion had hypokalaemia (serum potassium <3.5 mmol/L; Table 1; Fig. 3) [9]. Few participants were hospitalized for hyperkalaemia (≤ 0.3%) and the proportion hospitalized for hyperkalaemia was similar between eplerenone and placebo [9]. The clinical benefits of eplerenone in EMPHASIS-HF were observed independently of the occurrence of hyperkalaemia [48].

Finerenone was associated with an approximately 2-fold higher incidence of serum potassium > 5.5 mmol/L compared with placebo in FINEARTS-HF (Table 1) [11, 23]. Investigator-reported hyperkalaemia was higher in the finerenone group than the placebo group [11]. To manage this known risk of MRA therapy, the dose of finerenone in the FINEARTS-HF trial was based on baseline eGFR; in participants with an eGFR of ≤ 60 mL/min/1.73 m^2^, finerenone dose was titrated to half of that in participants with higher eGFR [11, 23]. While the absolute incidence of serum potassium > 5.5 mmol/L was higher in participants with lower eGFR, the relative risk of serum potassium > 5.5 mmol/L between finerenone and placebo remained consistent across target doses [23]. The incidence of hyperkalaemia leading to hospitalization was low and there were no hyperkalaemia-related deaths [11, 23]. Higher potassium levels at baseline, higher serum creatinine and UACR, and type 2 diabetes were all associated with a greater risk of serum potassium >5.5 mmol/L during the study [23]. The incidence of serum potassium < 3.5 mmol/L was lower with finerenone compared with placebo [11, 23]. Both potassium > 5.5 mmol/L and < 3.5 mmol/L increased the subsequent risk of the primary outcome in both the finerenone and placebo groups [23]. The benefits of finerenone, relative to placebo, on the primary outcome were observed even in participants who experienced hyperkalaemia post-randomization [23].

In a meta-analysis of phase 3 MRA studies (RALES, EMPHASIS-HF, TOPCAT, FINEARTS-HF), the risk of hyperkalaemia was twice as high in participants treated with an MRA compared with those receiving placebo, irrespective of EF; however, the absolute risk of serious hyperkalaemia (serum potassium > 6 mmol/L) with MRA was low, ranging from 2% to 4% [71]. This meta-analysis also showed that the risk of hypokalaemia was halved with MRA therapy compared with placebo [71].

While there have been no large-scale trials comparing MRAs to each other, in a pilot study that compared finerenone (20–40 mg/day) and spironolactone (20–40 mg/day) in 60 patients with primary aldosteronism, finerenone was associated with a smaller increase in serum potassium levels at day 56 compared with spironolactone (mean increase, 0.2 [standard deviation, 0.4] mmol/L vs. 0.5 [0.4] mmol/L; *p <* 0.05) [60]. In the phase 2b ARTS-HF study, in participants with worsening HF and diabetes or CKD, the incidence of hyperkalaemia was 4.7% with eplerenone (average daily dose 38.6 mg), and 3.6% and 6.3% in participants uptitrated to 20 mg once daily finerenone from a starting dose of 10 mg and 15 mg once daily, respectively [66].

### Practical considerations in MRA implementation

**Fig. 4:**
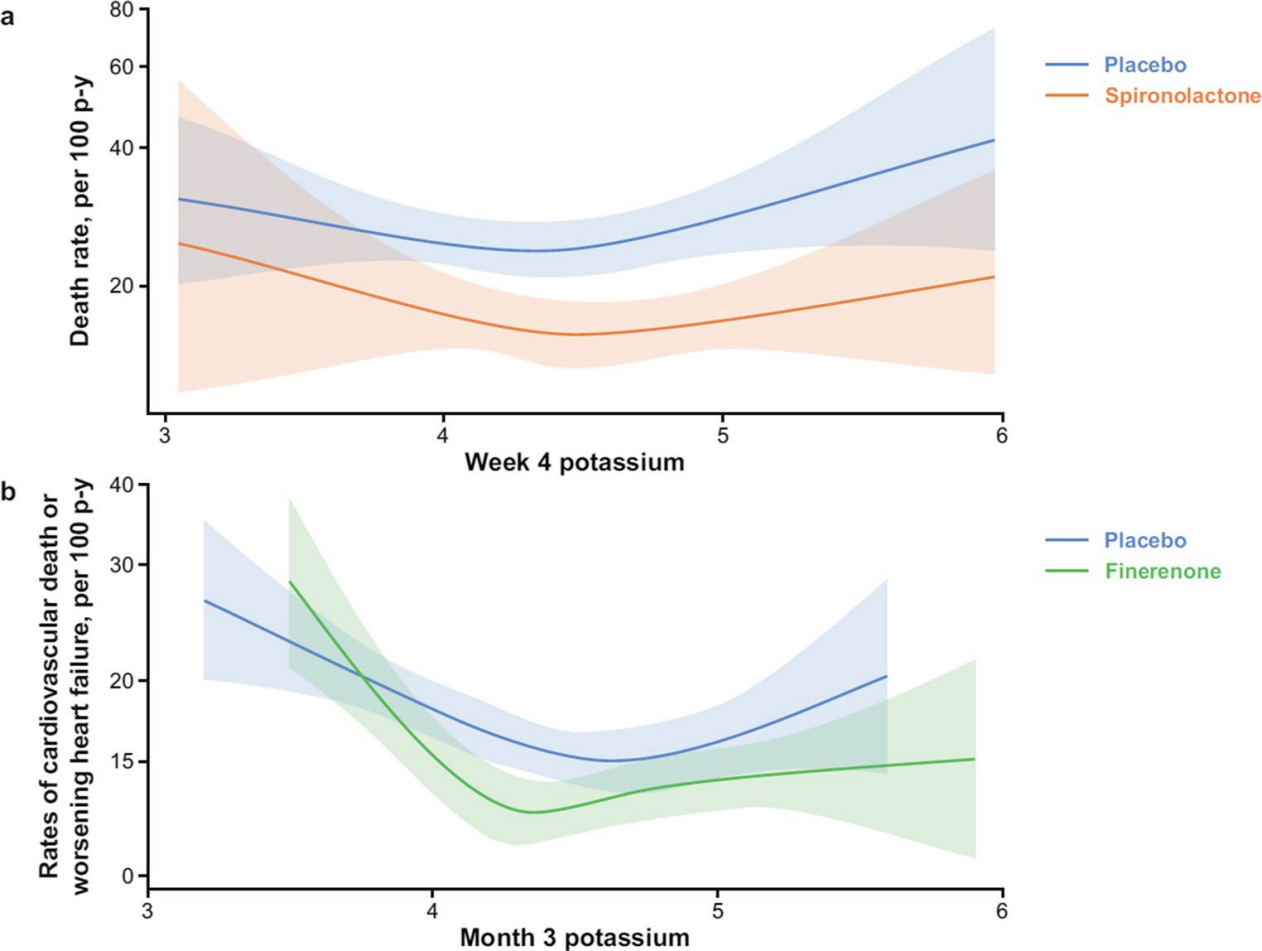
Event rates by treatment according to mean serum potassium levels in (mmol/L): (**a**) RALES, (**b**) FINEARTS-HF. *p-y* person-years. Part A reproduced with permission from Vardeny et al. 2014 [70]. Part B reproduced from Vardeny et al. 2025 [23] under the terms of the CC-BY-NC-ND 4.0 license

The data from phase 3 studies of MRAs in participants with HF, discussed above, demonstrate a favourable benefit–risk profile for the use of MRAs in individuals with HF across the EF continuum. An increased risk of high serum potassium levels is common in HF due to disease progression, comorbidities, and the use of medications that alter potassium homeostasis [23, 72]. Concerns about the risk of hyperkalaemia may lead to the underuse of MRAs in HFrEF in clinical practice; however, MRAs are effective for improving outcomes even when potassium levels are moderately elevated (Fig. 4) [23, 48, 70] and in individuals with comorbid CKD [45]. The risk of adverse outcomes associated with hypokalaemia may be underappreciated [23], and the clinical benefits of MRAs in individuals with HF may be partly attributable to the lower incidence of hypokalaemia with MRAs [23, 73].

Based on study design protocols from phase 3 trials with MRAs, one strategy for reducing the risk of hyperkalaemia in individuals with HF treated with MRAs in clinical practice is appropriate drug dosing. Risk factors for hyperkalaemia, such as lower eGFR, higher baseline serum potassium, diabetes, and lower haemoglobin (Table 3) [22, 31, 32, 74, 75], should be incorporated into treatment protocols to ensure appropriate dose initiation and adjustment. As in the FINEARTS-HF trial [11], finerenone dosing could be informed by baseline kidney function; individuals with an eGFR of ≤ 60 mL/min/1.73 m^2^ should have their dose initiated and titrated to half that used for participants with a higher eGFR. A similar strategy can be adopted for sMRAs, as was employed in the EMPHASIS-HF study [9]; the starting dose of eplerenone could be initiated at 25 mg every other day for participants with an eGFR of < 50 mL/min/1.73 m^2^ and 25 mg daily for those with a baseline eGFR of ≥ 50 mL/min/1.73 m^2^. In addition, thresholds for intervention and algorithms could minimize unnecessary treatment discontinuations. In MRA trials, these included regular monitoring of serum potassium, MRA therapy downtitration, temporary discontinuation, and safe reinitiation following hyperkalaemia events [9, 23].

**Table 3:** Risk factors for hyperkalaemia

Risk factor [22, 31, 32, 74, 75]
Decreased kidney function
Higher baseline serum potassium
Diabetes
Proteinuria
Lower haemoglobin
Concomitant use of:
Potassium supplements
Potassium-sparing diuretics
Angiotensin-converting enzyme inhibitors
Angiotensin receptor blockers
Nonsteroidal anti-inflammatory drugs
Cytochrome P450 3 A inhibitors (including grapefruit juice)
Cholestyramine
Sulphonamide antibiotics
Herbal preparations (turmeric, Siberian ginseng)

Certain medications (including those available over the counter, such as nonsteroidal anti-inflammatory drugs and some herbal preparations), may contain potassium or raise potassium levels [75], and should be avoided to mitigate the risk of hyperkalaemia (Table 3). In contrast, potassium binders may be used to reduce the risk of hyperkalaemia in people treated with MRAs, as has been shown in clinical trials of spironolactone [76].

Learnings from trial analyses in patients with type 2 diabetes and CKD could guide care. Concomitant use of SGLT2is and sMRAs in people with type 2 diabetes and CKD is associated with a reduced incidence of hyperkalaemia compared with sMRAs alone [77], and a similar reduction has been observed in patients with HF [78]. However, in the CONFIDENCE trial in people with type 2 diabetes and CKD, there were numerically higher mean serum potassium values with combined use of an SGLT2i and finerenone than with finerenone alone; the difference was not statistically significant and there was a lower incidence of hyperkalaemia with combined therapy than with finerenone alone [18, 79]. Risk factors for hyperkalaemia included higher baseline potassium, lower eGFR, higher albuminuria, and more advanced CKD stage [79, 80]; these factors should inform more judicious dosing at the point of care.

Currently, the American Heart Association (AHA)/American College of Cardiology (ACC)/Heart Failure Society of America (HFSA) 2022 [1] and European Society of Cardiology (ESC) 2021 [2] guidelines have a class 2B recommendation for the use of sMRAs in HFmrEF. The AHA/ACC/HFSA 2022 [1] guidelines also recommend sMRAs (class 2B) for use in HFpEF. As of April 2026, these guidelines have not been updated to include finerenone, which has a stronger recommendation than sMRAs in the Japanese, but not the Canadian, guidelines [20, 29]. In patients with diabetes and kidney disease, a comorbidity common in HF that is often associated with MRA underutilization [16, 41], nsMRA has a class I indication [3, 81]. No phase 3 trials have directly compared the efficacy and safety of sMRAs and finerenone to date [82].

## Conclusion

High-quality evidence supports the use of sMRAs in HFrEF [1, 2, 29, 31, 32], and nsMRAs in HFmrEF and HFpEF [1, 3, 10, 11]. Several clinical trials also demonstrate the efficacy of nsMRAs in the treatment of diabetes with kidney disease and the prevention of heart failure [14, 15, 17, 26]. The increased risk of hyperkalaemia with all MRAs, particularly in patients with high albuminuria, low eGFR, and advanced CKD stage [22, 32, 75], limit their use in patients who are likely to derive the greatest benefits from treatment [82]. It is important to note that the benefits of MRAs are retained despite moderately elevated serum potassium levels [7–9, 11, 12, 23, 70, 71] and that risk can be mitigated by using lower doses and avoiding the concomitant use of medications that increase potassium [31, 32, 74]. Conversely, MRAs are associated with a lower incidence of hypokalaemia [8, 9, 11, 21–23, 71], which may contribute to their clinical benefits in individuals with HF [23, 73]. Alongside concomitant use of SGLT2 inhibitors and/or potassium binders, and with appropriate strategies for monitoring serum potassium levels and adjusting dosages accordingly, the greater use of MRAs in eligible patients with HF will improve outcomes.

## Data Availability

No datasets were generated or analysed during the current study.
